# An Acceptance and Commitment Therapy Prototype Mobile Program for Individuals With a Visible Difference: Mixed Methods Feasibility Study

**DOI:** 10.2196/33449

**Published:** 2022-01-21

**Authors:** Fabio Zucchelli, Olivia Donnelly, Emma Rush, Paul White, Holly Gwyther, Heidi Williamson, The VTCT Foundation Research Team at the Centre for Appearance Research

**Affiliations:** 1 Health and Applied Sciences University of the West of England Bristol United Kingdom; 2 North Bristol National Health Service Trust Bristol United Kingdom; 3 Vitiligo Support United Kingdom London United Kingdom; 4 See Authors' Contributions

**Keywords:** mobile health, acceptance and commitment therapy, appearance, mixed methods, mobile phone

## Abstract

**Background:**

Mobile apps may offer a valuable platform for delivering evidence-based psychological interventions for individuals with atypical appearances, or visible differences, who experience psychosocial appearance concerns such as appearance-based social anxiety and body dissatisfaction. Before this study, researchers and stakeholders collaboratively designed an app prototype based on acceptance and commitment therapy (ACT), an evidence-based form of cognitive behavioral therapy that uses strategies such as mindfulness, clarification of personal values, and value-based goal setting. The intervention also included social skills training, an established approach for increasing individuals’ confidence in managing social interactions, which evoke appearance-based anxiety for many.

**Objective:**

In this study, the authors aim to evaluate the feasibility of an ACT-based app prototype via the primary objectives of user engagement and acceptability and the secondary feasibility objective of clinical safety and preliminary effectiveness.

**Methods:**

To address the feasibility objectives, the authors used a single-group intervention design with mixed methods in a group of 36 participants who have a range of visible differences. The authors collected quantitative data via measures of program use, satisfaction ratings, and changes over 3 time points spanning 12 weeks in outcomes, including selected ACT process measures (experiential avoidance, cognitive defusion, and valued action), scales of appearance concerns (appearance-based life disengagement, appearance-fixing behaviors, appearance self-evaluation, and fear of negative appearance evaluation), and clinical well-being (depression and anxiety). Semistructured exit interviews with a subsample of 12 participants provided qualitative data to give a more in-depth understanding of participants’ views and experiences of the program.

**Results:**

In terms of user engagement, adherence rates over 6 sessions aligned with the upper boundary of those reported across mobile mental health apps, with over one-third of participants completing all sessions over 12 weeks, during which a steady decline in adherence was observed. Time spent on sessions matched design intentions, and engagement frequencies highlighted semiregular mindfulness practice, mixed use of value-based goal setting, and high engagement with social skills training. The findings indicate a good overall level of program acceptability via satisfaction ratings, and qualitative interview findings offer positive feedback as well as valuable directions for revisions. Overall, testing for clinical safety and potential effectiveness showed encouraging changes over time, including favorable changes in appearance-related life disengagement, appearance-fixing behaviors, and selected ACT measures. No iatrogenic effects were indicated for depression or anxiety.

**Conclusions:**

An ACT-based mobile program for individuals struggling with visible differences shows promising proof of concept in addressing appearance concerns, although further revisions and development are required before further development and more rigorous evaluation.

## Introduction

### Background

Visible difference refers to an unusual physical appearance caused by a congenital or acquired health condition, injury, or medical intervention [1]. Between visible skin conditions such as psoriasis and eczema, scarring from trauma or surgery, craniofacial conditions such as cleft lip and/or palate, and many other causes, approximately 1 in 60 people are estimated to live with a visible difference [2]. In the context of heightened conspicuousness and common reports of negative social reactions [3], there is a marked prevalence of appearance-related distress, most commonly in the form of anxiety (particularly social anxiety, marked by a fear of negative appearance evaluation and social withdrawal), depression, and body dissatisfaction, across a range of appearance-affecting conditions [4-6]. Although many adjust well to having a visible difference, samples of individuals with visible differences commonly show significantly higher rates of appearance-based anxiety and depression across cultures (eg, in adults with acne [7] and alopecia [8]) when compared with matched controls. With minimal specialist psychological provision available (eg, across Europe [9]), the development and testing of specialist self-help interventions are warranted. Indeed, existing self-help shows promise in addressing appearance concerns for this population [10].

### Acceptance and Commitment Therapy for Visible Differences

Acceptance and commitment therapy (ACT) offers a novel approach to the population with visible differences, with traditional cognitive behavioral therapy having dominated the research field to date [11]. ACT is a third-wave cognitive behavioral therapy in which psychological flexibility is cultivated, namely, the capacity to hold present moment awareness and acceptance of one’s internal experiences while acting in ways aligned to one’s personal values [12]. The psychological flexibility model holds conceptual promise for addressing appearance concerns in this group, for some of whom thoughts such as “I look strange” or “People will stare” represent a degree of objective reality, therefore being less amenable to the thought challenging approaches adopted in traditional cognitive behavioral therapy. In ACT, individuals learn to accept the presence of such unwanted thoughts and associated emotions while also detaching from their literality through an awareness of thoughts as an ongoing internal process rather than focusing on their content (via *cognitive defusion*). Concurrently, individuals clarify their personal values as guides for ongoing purposeful behavior. These values are qualities of behavior, namely, a quality that can be enacted, such as skillfulness, honesty, and intimacy. Present moment acceptance and cognitive defusion are cultivated in ACT primarily as a means of facilitating a commitment to value-consistent activity. In combination, ACT may offer a pragmatic therapeutic approach for this population [13].

In a cross-sectional study involving individuals with a range of visible differences, researchers [14] drew from an established model of body image coping strategies [15] to investigate the role of 2 key psychological flexibility subprocesses in relation to appearance-related outcomes. The authors found that cognitive fusion (taking thoughts as factual statements to be acted on) mediated the relationship between appearance evaluation and 2 unhelpful coping strategies in the form of appearance-related life disengagement (physically avoiding situations because of appearance concerns) and appearance-fixing behaviors (eg, compulsive concealment of the visible difference). They also found that experiential avoidance (an unwillingness to remain in contact with unwanted internal experiences) partially mediated disengagement but not appearance-fixing behaviors. Cognitive fusion, experiential avoidance, and reduced committed action were also found to correlate with appearance anxiety in patients with burn scarring [16].

Recent trials offer some evidence for the efficacy of book-based ACT self-help in related clinical areas of social anxiety [17] and body dissatisfaction in the general population [18]. These interventions that are based on evidence-based ACT protocols [19,20] offer a valuable starting point for developing ACT programs for individuals with visible differences. However, modification is needed both from social anxiety interventions (eg, acknowledging that individuals with visible differences may encounter initial negative reactions to their appearance from others) and body dissatisfaction protocols, which predominantly focus on shape and weight rather than unusual appearance. To the authors’ knowledge, no research investigating any other ACT self-help interventions for individuals with visible differences has been published.

### Mobile Delivery

Mobile apps offer a unique level of user functionality to facilitate everyday skills training and self-monitoring in self-help programs [21]. Real-time tailored feedback can reinforce target behaviors [22], which, for individuals with a visible difference, could include the practice of social skills training (an evidence-based component to help individuals manage difficult social encounters [23]), tailored mindfulness practices, and valued goals related to appearance concerns. Notifications can also be set to remind users to engage, a function that has been shown to improve the efficacy of mental health apps [24]. In addition to acting as standalone interventions for individuals with mild to moderate support needs, apps can be used to augment professional interventions [25,26].

Research on existing ACT-based app interventions offers a valuable direction for designing ACT-based apps for behavior change. For example, participants who used a more complex ACT matrix health app made greater health improvements and used the app more than those who used a simpler version [27], suggesting that users may prefer a variety of novel toolbox-like activities. The involvement of key stakeholders at the design stage of an app program, including end users from the intended population and clinicians with relevant expertise, is also paramount to optimize its appeal, accessibility, and trustworthiness [28]. For this reason, the intervention investigated in this study was designed using participatory design methods involving individuals with visible differences and clinicians with relevant expertise, as detailed in a previous article [29].

Another design consideration is whether to make a mobile app self-guided or guided. In general, higher levels of professional input may confer greater efficacy [24]. However, self-guided mobile apps require fewer support resources and, therefore, are capable of reaching more users. Equally, in the field of ACT self-help studies, evidence for the superiority of guided interventions is equivocal. Selvi et al [18] found no additional benefit of guided self-help for body dissatisfaction compared with the self-guided version. Similarly, in a trial of an ACT-based social anxiety intervention delivered on the web and via an app (with equivalent content), researchers [30] found no differences between guided and unguided versions of the intervention on social anxiety outcomes. The authors proposed that mobile app features may partially compensate for the absence of a guiding therapist, for example, by providing real-time feedback during exposure tasks. However, an absence of professional oversight may heighten concerns of potential iatrogenic effects on well-being [31]. Therefore, it is important to mitigate against and test for any iatrogenic effects in the design and testing of mobile interventions.

### Goal of This Study

The aim of this study is to assess the feasibility, or proof of concept, of an ACT-based self-guided prototype mobile intervention for individuals with visible differences who experience appearance-related concerns. The prototype is delivered via a mobile-optimized web app that simulates a native mobile app, which is consistent with the recommendation to test low-cost iterations of behavior change apps before building full-scale versions [32]. By assessing the prototype’s feasibility using a mix of quantitative and qualitative methods, we aim to make subsequent modifications to develop a native app and test this via a future randomized controlled trial (RCT).

The primary feasibility objectives target user engagement and acceptability, and the secondary objective is to determine whether the program indicates clinical safety and preliminary effectiveness.

## Methods

### Participants

A sample of 36 adults was recruited between July and November 2020. The primary recruitment strategy drew from 19 UK charities that represented and supported individuals with a range of congenital and acquired appearance-affecting conditions and advertised the study to potential participants via social media, newsletters, distribution lists, and web-based events. Researchers also promoted the study on relevant Reddit subgroups with the aim of boosting the number of male participants, given the comparatively higher use of Reddit by men versus women [33].

To be eligible, participants had to self-report as having a visible difference (defined to participants as a physical appearance they considered to be significantly different from a typical appearance, with a list of example causes given). Eligibility also included currently experiencing appearance concerns, defined as psychological and/or social difficulties related to their visible differences, such as appearance-based social anxiety, low mood, and body image concerns. Participants also had to be aged ≥18 years, a UK resident, own a smartphone and have regular internet access (either through home Wi-Fi and/or cellular data), and have experience in using apps. Participants were ineligible if they acquired a visible difference from traumatic injury in the preceding 6 months because of the heightened risk of unprocessed psychological trauma, for which a self-administered intervention would be clinically inappropriate. Ineligibility also included current experience of a mental health crisis (eg, suicidality or self-harm), undergoing talking therapy, or having appearance concerns primarily related to weight or eating. The sample’s mean age was 36.67 (SD 14.25) years. All other demographic characteristics are presented in Table 1.

**Table 1:** Demographic characteristics of total study sample (N=36).

Characteristics			Values, n (%)
Gender (female)			29 (81)
**Cause of visible difference**			
	Skin condition (eg, alopecia, ichthyosis, psoriasis, eczema, and scarring)	20 (56)	
	Congenital craniofacial condition (eg, cleft lip and/or palate and craniosynostosis)	6 (17)	
	Other congenital conditions (eg, birthmark and inherited ichthyosis)	7 (19)	
	Acquired craniofacial condition (eg, facial palsy and malocclusion of jaw)	3 (8)	
**Ethnicity and race**			
	White	32 (89)	
	Mixed ethnic groups	2 (6)	
	Asian or Asian British	1 (3)	
	Black, African, or Caribbean	1 (3)	
**Relationship status**			
	Single	15 (42)	
	Married or in a civil partnership	9 (25)	
	Dating or living with a partner	9 (25)	
	Separated or divorced	2 (7)	
	Would rather not say	1 (3)	
**Occupation status**			
	Employed full time	16 (44)	
	Employed part time	6 (17)	
	Student	5 (14)	
	Retired	3 (8)	
	Unemployed	3 (8)	
	Unable to work	3 (8)	
**Highest education level**			
	Graduate degree	10 (28)	
	Undergraduate degree	10 (28)	
	Vocational qualification	10 (28)	
	High school	6 (17)	

### The Intervention

The program *ACT It Out* comprises 6 sessions designed to take approximately 30 minutes each and be completed weekly, augmented with between-session *Skill builder* toolbox-like activities for the everyday practice of the ACT-based skills presented in session. Textbox 1 shows an overview of the program content. The material was derived from a combination of the intervention research literature and stakeholder input. ACT-specific content was drawn from evidence-based protocols for social anxiety [19] (eg, *safety mode* for threat-focused attention in public) and body dissatisfaction [20] (eg, *mindful mirror exercise*) and adapted for the population, for example, by acknowledging the possibility of unsolicited public attention in relation to social anxiety and modifying the mindful mirror script to account for participants’ bodily areas of visible difference rather than their body weight and shape. Social skills training for managing difficult social interactions was informed by existing evidence-based self-help programs, for example, the study by Bessell et al [23].

**Overview of *ACT It Out* program content.**

**Session 1**

- What *ACT It Out* is and how it works
- Choice point metaphor (toward and away moves); simple reflection on personal values
- Passengers on a bus metaphor

**Postsession 1: Skill builders**

- Recording your passengers and toward or away moves

**Session 2**

- Reviewing your passengers and toward or away moves
- Responding to your passengers (acceptance and commitment therapy social anxiety concepts of safety mode versus *ACT It Out* mode)
- Mindfulness training—your senses

**Postsession 2: Skill builders**

- Micromindfulness (text instructions)

**Session 3**

- Reviewing safety mode versus *ACT It Out* mode
- Values and taking action (values clarification and setting a simple goal for the day)
- Mindfulness training—breath and body

**Postsession 3: Skill builders**

- Tracking simple goals for the day
- Mindfulness of breath and body

**Session 4**

- Reviewing valued action (setting a simple goal for the week) and mindfulness
- Mindfulness training—breathing into intensity
- Being around people—taking control (social skills)

**Postsession 4: Skill builders**

- Tracking simple goals for the week
- Mindfulness of intense experiences
- Social skills practice

**Session 5**

- Reviewing your goal and mindfulness
- Being around people—managing negative reactions (social skills)
- Mindfulness training—mindful mirror

**Postsession 5: Skill builders**

- Social skills practice
- Mindful mirror

**Session 6**

- Reviewing your mindfulness and social skills
- Mindfulness in daily life
- *ACT It Out* long term (long-term goal setting)

**Postsession 6: Skill builders**

- Tracking long-term goals

*ACT It Out* underwent an iterative design process led by the first author (FZ), incorporating feedback from user representatives and psychological practitioners with expertise in ACT and/or visible differences. The second and third authors (OD and ER) contributed to the program’s design and testing as clinical lead and user representative lead, respectively. Examples of user-driven content incorporated into the prototype included a *human* interactive element (involving an *app guide*, the second author OD); real case examples throughout to illustrate ACT principles and offer a sense of commonality (many provided by user representatives); and accessible formatting, including large font, subtitles, and audio transcripts. Content based on clinicians’ input reflected their preferences for the inclusion of a range of established ACT metaphors used to convey important concepts, management of users’ expectations through clear information and periodic *check-ins* on the ACT model (eg, reiterating that the goal is not to directly reduce negative affect), and provision of sufficient time for behavior modification.

*ACT It Out* is self-guided, although the in-built *app guide* featuring in the introductory videos in each session and in tailored feedback throughout is designed to facilitate human interaction. ACT metaphors integrated into the program included *passengers on a bus* (whereby unwanted thoughts and feelings are represented by passengers on a bus), the *choice point* (in which a dichotomous choice is presented between actions that serve personal values (*toward moves*) and those that do not (*away moves*) [34]), and modified psychological flexibility *pillars* (with the different psychological flexibility subprocesses each presented as a pillar) that can be built up over time, with the addition of self-compassion, as used in clinical practice for this population by the second author (OD) [18]. These were presented in a multimedia form via videos, pictures, and interactive exercises (as preferred by ACT clinicians [25]), along with purpose-made guided mindfulness practices. Self-monitoring of various ACT processes via single questions (eg, present moment contact), value-based goals, and social skills training was integrated throughout. Real-time behavioral recording (eg, of *toward* or *away moves*) was built into *Skill builders*, and personalized timed reminders to engage in *Skill builders* and subsequent sessions were programmed.

As new content was introduced in each session, users were encouraged to complete all 6 sessions, which would represent full completion. However, the core components of the program in the form of mindfulness (incorporating acceptance and cognitive defusion), self-compassion, value clarification, value-based goal setting, and social skills were all covered by session 4, with sessions 5 and 6 building on and consolidating these components. Completion of sessions 1 to 4 could therefore be expected to represent a cutoff for minimal completion.

**Figure 1:**
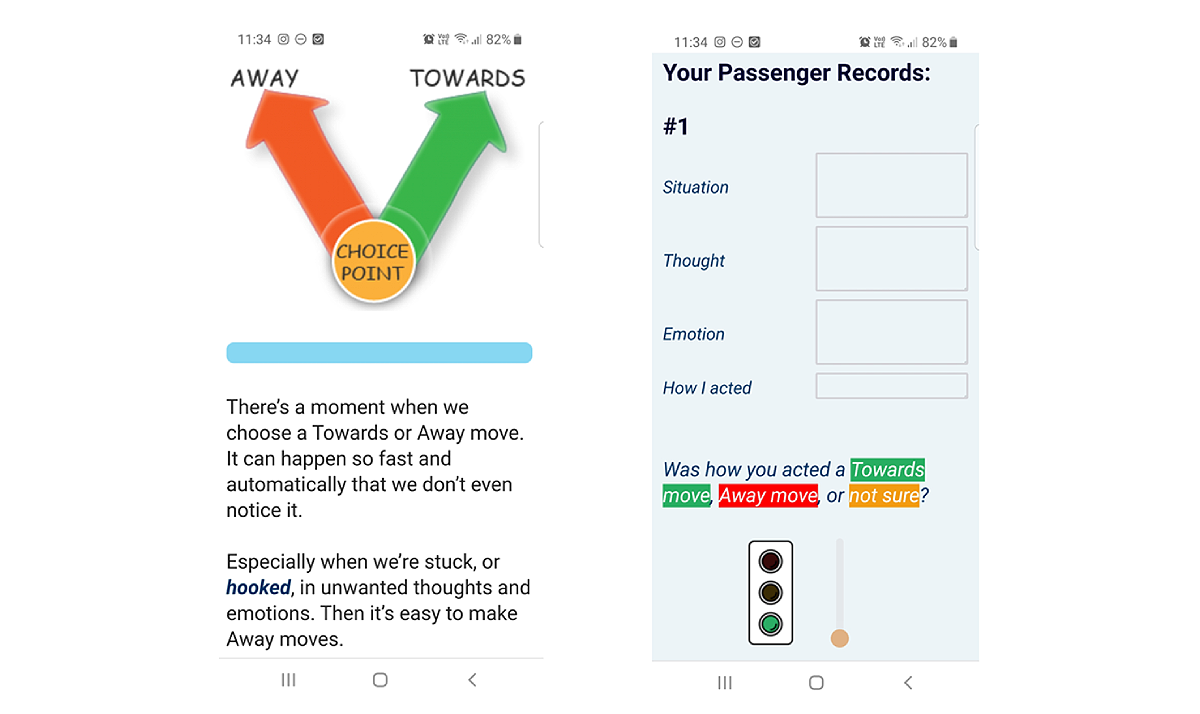
Example *ACT It Out* screenshots.

Program content was delivered via the Qualtrics XM (Qualtrics International Inc) survey web app, which was constructed by the first and fifth authors (FZ and HG). Qualtrics XM is a mobile-optimized survey platform that can accommodate many native app features, such as embedded multimedia, responsive content tailored to users’ actions, and discrete *toolbox* options accessible from the home screen. Example screenshots are shown in Figure 1. Text reminders were programmed to simulate push notifications.

### Design

This study adopted a single-group mixed methods design. We chose this design for its suitability to the study’s objectives of user engagement, acceptability, and clinical safety and preliminary effectiveness, which together focused on assessing proof of concept before justifying native app development and more rigorous testing of a final product via a randomized design [35]. These objectives and the study design were informed by a combination of the Bowen et al [36] feasibility study design recommendations, as used in comparable mobile app feasibility trials [37,38]; the UK Medical Research Council guidance on developing and testing complex interventions [39,40]; and the Obesity-Related Behavioral Intervention Trials model for developing behavioral interventions in long-term health conditions [35]. As is the case in this study, both the UK Medical Research Council and Obesity-Related Behavioral Intervention Trials models recognize that there is often an overlap between the later objectives of the development phase, in which an intervention is refined, and the early feasibility objectives of the testing phase.

Led by this guidance, the authors used a range of mixed methods to meet the objectives. The authors aimed to assess user engagement via program adherence, duration of use, and in-program use. Acceptability was determined via satisfaction ratings and explored in greater depth through semistructured qualitative exit interviews.

To test the secondary objective of whether the program indicated clinical safety and preliminary effectiveness, we assessed changes in a range of outcomes over 3 time points. The outcomes included (1) appearance-related life disengagement and appearance-fixing behaviors as primary outcomes of targeted behavior change; (2) measures of key targeted ACT processes, cognitive fusion, and experiential avoidance, as indicated in prior research [14], as well as valued action (a targeted behavioral component of psychological flexibility); and (3) clinical well-being outcomes and secondary appearance-focused measures to test for iatrogenic effects. Clinical safety would be indicated by improvement or stability in the relevant measures and preliminary effectiveness indicated by improvement in measures. Completion rates and scale attenuation effects were also checked for measures to assess their feasibility for use in future RCTs. In addition, qualitative interviews were used to gain a richer understanding of how participants may have experienced the program in terms of perceived psychological safety and any benefits from using the program.

### Procedure

Ethical approval was granted by the university faculty research ethics committee. The first author (FZ) contacted interested individuals by their chosen method, then arranged a 15- to 20-minute orientation telephone call, in which FZ confirmed individuals’ eligibility, guided participants in setting up the program, and oriented them to the platform. Before commencing the program, participants were instructed to check and complete a web-based consent checklist, followed by baseline outcome measures. Upon consent, participants were sent a £10 (US $13.28) web-based shopping voucher to compensate for any data use costs. Outcome measures were sent to participants after 8 weeks (allowing 2 additional weeks for anticipated time slippage) and after 12 weeks, when a second compensatory £10 (US $13.28) web-based shopping voucher was sent to completers. Access to *ACT It Out* was available to participants for 12 weeks as the authors sought to measure completion duration as part of the user engagement objective.

**Figure 2:**
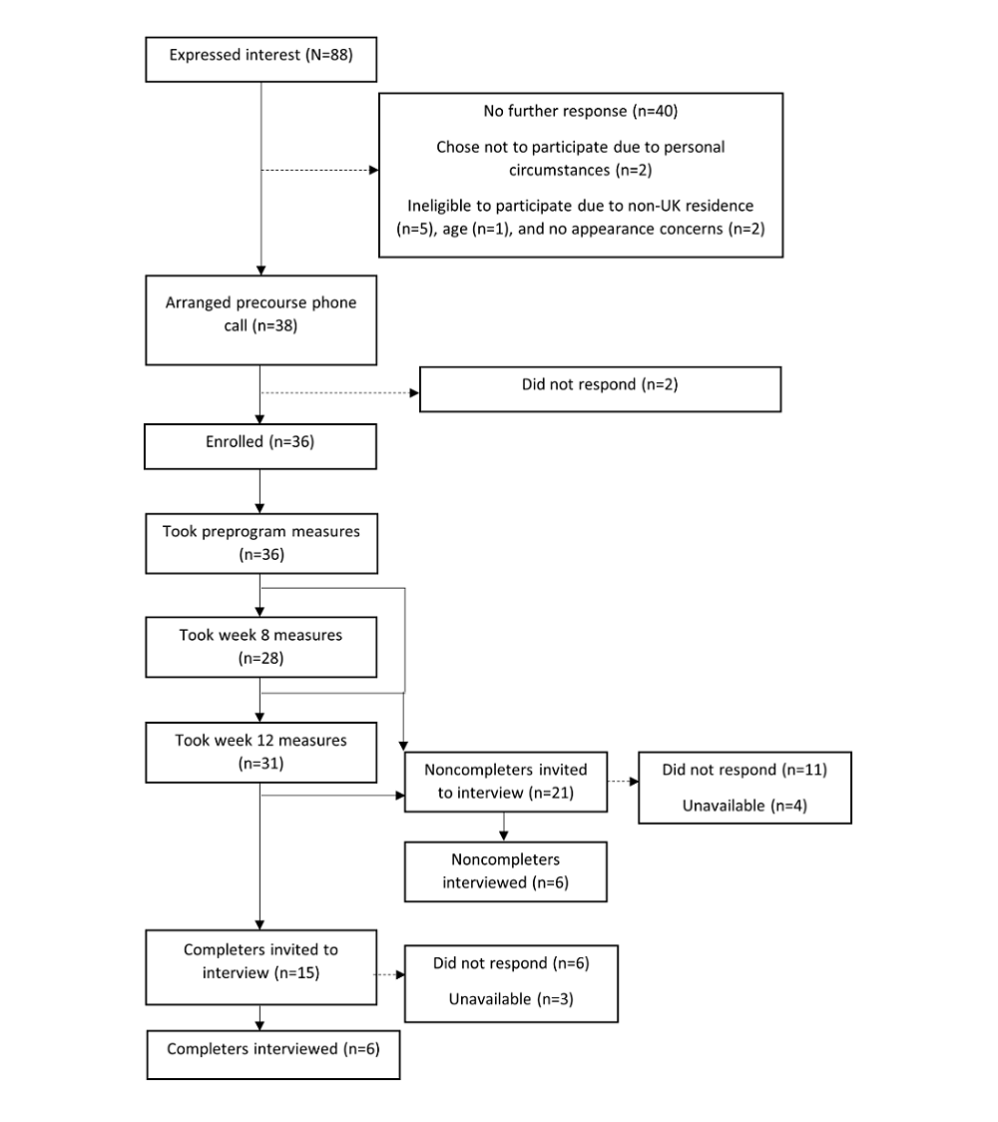
Diagram of participant flow, dropout, and reasons for exclusion (where N refers to the number of potential participants and n refers to a subsample of the population under study).

Semistructured exit interviews with FZ were planned for a representative subsample of up to half of the sample, including program noncompleters. Full completers were invited after the 12-week measurement, and noncompleters were invited 3 weeks after their final use of *ACT It Out* to mitigate loss in recall memory. Participants were invited via email, with the information sheet sent as an attachment. The first author (FZ) took verbal consent at the start of the interview and sent participants a £10 (US $13.28) web-based shopping voucher following the interview (offered as a small incentive). Participant flow and dropout across the entire study are presented in Figure 2.

This study coincided with the COVID-19 pandemic. In consultation with the user representative lead (third author, ER), we decided to commence the study in July 2020 when national COVID-19 *lockdown* restrictions had been eased, and study recruitment was ended when a further national lockdown was enforced in November 2020.

### Measures

#### Quantitative Measures

##### User Engagement

Data embedded within the program were collected on total and session-by-session duration of use, as well as self-reported engagement with key content features, including value-based goals and mindfulness and social skills exercises. Session-by-session program adherence was monitored and recorded daily by the first author (FZ).

##### Acceptability

Satisfaction rating questions were embedded at the end of each program session, with ratings ranging from 1 (*strongly disagree*) to 5 (*strongly agree*). These questions were based on a previous feasibility trial of a web-based intervention for individuals with visible differences [41]. Participants’ mean scores across all completed sessions were calculated to provide an overall satisfaction score.

##### Clinical Safety and Preliminary Effectiveness Appearance Measures

The Body Image Life Disengagement Questionnaire [42] was used to measure the degree of participants’ disengagement from appearance-salient activities across recreational, social, and occupational life domains because of appearance concerns. Items are rated from 1 (*has not stopped me at all*) to 4 (*stopped me all the time*). The 11-item modified version of the 10-item original version that includes a population-specific item (*use public transport*) has been used in a previous study with visibly different participants, showing good reliability [14]. Reliability in this study was good (Cronbach α=.88). The score range of the Body Image Life Disengagement Questionnaire is 1 to 4, with a lower mean score indicating greater life engagement.

The 10-item Body Image Coping Strategies Inventory–Appearance-fixing (BICSI-AF [15]) subscale assessed participants’ tendency to conceal, correct, seek reassurance, and engage in social comparisons about their appearance. Items are rated between 0 (*definitely not like me*) and 3 (*definitely like me*). The BICSI-AF demonstrated good reliability and validity in college students [15] and strong reliability in visibly different populations [14,43]. Internal consistency in this study was good (Cronbach α=.86). The score range of the BICSI-AF is 0 to 3, with higher mean scores representing greater use of appearance-fixing behaviors.

The Body Esteem for Adolescents and Adults–Appearance subscale [44] measured participants’ evaluation of their own appearance on 10 items. The subscale has shown good reliability and validity in young adults [45] and good reliability in samples with visible differences [14,46]. In this study, reliability was excellent (Cronbach α=.92). A total of 6 items were reverse coded, and higher scores in the range of 0 to 4 indicated a more positive appearance evaluation.

The 6-item Fear of Negative Appearance Evaluation Scale (FNAES [47]) was used to assess participants’ level of concern that others would negatively evaluate their appearance. The FNAES has been shown to have good validity and reliability in college students [47], and reliability in this study was excellent (Cronbach α=.96). The scale range for the FNAES is 6 to 30, with higher scores suggesting participants’ greater level of concern.

##### ACT Measures

The Brief Experiential Avoidance Questionnaire [48] measured participants’ level of experiential avoidance using 15 items. The scale showed strong reliability and validity in a mixed adult group [48], good internal consistency in a previous study with visibly different participants [46], and good internal consistency in this study (Cronbach α=.86). The Brief Experiential Avoidance Questionnaire scores range from 15 to 90, with higher scores indicating higher experiential avoidance.

The 7-item Cognitive Fusion Questionnaire (CFQ [49]) assessed participants’ degree of cognitive fusion. The CFQ has good validity and reliability in clinical and nonclinical adult samples [49] and showed strong reliability in a visibly different population [14]. In this study, reliability was good (Cronbach α=.86). The score range of the CFQ is 7 to 49, with higher scores representing higher cognitive fusion.

The Comprehensive Assessment of Acceptance and Commitment Therapy–Valued Action subscale [50] was used to determine the extent to which participants engaged in value-oriented action, combining the traditional psychological flexibility subprocesses of value clarification and committed action. The 8-item subscale demonstrated strong reliability and validity in a variety of adult samples [51] and showed acceptable reliability in this study (Cronbach α=.78). The Comprehensive Assessment of Acceptance and Commitment Therapy–Valued Action scores range from 0 to 48, with higher scores reflecting greater valued action.

##### Clinical Well-being Measures

The 14-item Hospital Anxiety and Depression Scale (HADS [52]) measured depression and anxiety in participants, with 7 items for each subscale. The HADS has well-established reliability and validity in a range of clinical and community populations [53]. In this study, internal consistency was acceptable for depression (Cronbach α=.77) and anxiety (Cronbach α=.74).

Anxiety and depression caseness on the scale range of 0 to 21 is indicated by subscale scores of 8 to 10 (mild), 11 to 14 (moderate), and 15 to 21 (severe). As the HADS alone is insufficient for a diagnosis, this study did not exclude participants scoring in the severe range. Instead, automated messages offering support contact details and a suggestion to consider whether involvement in the study was suitable were presented to any participants scoring in the severe range.

#### Qualitative Measures

To explore areas related to (1) program acceptability and (2) clinical safety and preliminary effectiveness in more depth, the first author (FZ) conducted semistructured interviews via telephone, lasting an average of 27 (SD 10.0; range 14-38) minutes. The schedule explored participants’ overall impression of *ACT It Out*, any aspects they particularly liked or disliked and/or found helpful or unhelpful, any notable benefits or detriments from using it, their views on the various program features, and, particularly for noncompleters, whether there were any aspects of the program that hindered adherence.

### Data Analysis

#### Quantitative Data

Frequencies and descriptive data (mean and SD) were calculated for data pertaining to user engagement and acceptability. Adherence was recorded in increments of 0.5 sessions to include partially completed sessions, which were defined as when participants completed a minimum of one-third of the relevant session without completing the full session. In exploratory analyses of the predictive effect of demographic data on user engagement, we used a binomial logistic regression model with age and education level (dichotomized into categories of higher education and high school or vocational training) as the independent variables and adherence (full completion or noncompletion) as the dependent variable. Independent sample *t* tests (2-tailed) were used to check for differences in acceptability scores by gender and education status, and Pearson correlation coefficients were calculated to test for a relationship between age and acceptability scores.

To assess clinical safety and preliminary effectiveness, mixed model repeated measure (MMRM) analyses were used to determine the significance level of any changes in scores between baseline and week 8, baseline and week 12, and across all time points. Hedge *g* was calculated as corrected effect sizes for baseline to week 8 and baseline to week 12 changes because of the small sample size [54]. Scale attenuation effects were checked for each scale at all time points by assessing the percentage of participants who reported the maximum and minimum possible scores, with ≥15% indicating ceiling or floor effects [55]. Of the 36 participants, 8 (22%) participants did not complete week 8 measures and 5 (14%) did not complete week 12 measures. The MMRM analyses accounted for all recorded data and modeled intention-to-treat analyses. Item-level missingness was negligible: a single item was missing from the data set, and available item analysis was used to score the scale in this case [56].

#### Qualitative Data

Interview data were analyzed by the first author (FZ) on NVivo software version 12 (QSR International) using thematic analysis from a primarily deductive approach; namely, themes were generated to answer the specific research questions of acceptability and preliminary effectiveness. The first author (FZ) followed the Braun and Clarke [57] six-step procedure, with minor modifications recommended by Braun and Clarke [58], as follows: (1) data familiarization through transcription, reading, and rereading; (2) generating initial codes across the entire data set; (3) generating draft themes; (4) reviewing themes by cross-referencing against coded extracts; (5) defining, refining, and naming themes; and (6) producing a report of the results and relating these findings to the research question and literature. The sixth author (HW) read a sample of interview transcripts and reviewed the first author’s (FZ) analysis. Minor changes to theme descriptions were made following discussions between the authors. FZ sent a summary of these findings to interview participants for the purpose of conferring trustworthiness via member checking [59]. Approximately 19% (7/36) of participants responded and requested no changes.

## Results

### User Engagement

**Figure 3:**
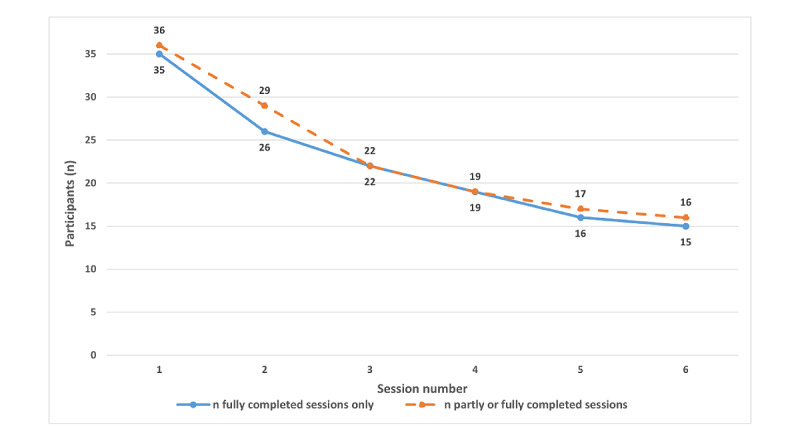
Session-by-session program adherence.

Out of the 6 sessions, participants completed an average of 3.32 (SD 1.85) sessions by week 8 and 3.72 (SD 2.11) by week 12. Of 36 participants, 16 (42%) completed the entire program by week 12, and 19 (53%) participants completed the suggested minimal completion cutoff of ≥4 sessions. Session-by-session adherence rates showed a slightly steeper dropout rate in the first half of the program (up to session 3), as shown in Figure 3.

A binomial logistic regression model with age and education level as predictors of adherence was statistically significant (*χ*^2^_2_=12.5; *P*=.002, with the combined predictors explaining 39.6% of the variance in the likelihood of participants completing the program (Nagelkerke *R^2^*). Within the model, age and education level were both statistically significant in isolation. Higher age of participants was associated with a greater likelihood of completing all 6 sessions, and those with a high school or vocational training level of education had 9.01 times higher odds of completing the program compared with those with university-level education. Owing to the low number of male participants, gender could not be included in the binomial logistic regression model. However, although based on a small number of male participants (7/36, 19%), the mean age of male participants was lower than that of the females (29/36, 81%; mean age: 25.57 years vs 39.34 years), and a lower proportion of males (2/7, 29%) fully completed the course compared with females (13/27, 48%), suggesting the possibility of a gender effect on completion likelihood, either in addition to or explaining the effect of age and education.

Participants spent an average of 25.7 (SD 14.67) minutes per session and 317.65 (SD 74.10) minutes in total over an average of 6.9 (SD 3.84) weeks. The engagement rates with value-based goal-setting activities are shown in Table 2. Of the 19 participants who completed session 4, 14 (74%) reported practicing the guided mindfulness of breath exercise introduced in session 3 *a few times (not daily)*; 3 (17%) reported practicing the exercise *daily*; and 2 (11%) reported *not once*. In the following session, of the 17 participants, 13 (76%) reported practicing the mindfulness of intense experiences exercise *a few times (not daily)*, and 2 (12%) selected *daily* and *not once*, respectively. Of the 16 participants who took session 6, 12 (75%) reported practicing the final guided mindfulness practices (mindful mirror) *a few times (not daily)*, 3 (19%) reported practicing it *daily*, and 1 (6%) selected *not once*. In session 6, participants were asked which of a set of statements best described their efforts in applying the social skills they had chosen to work on in sessions 4 and 5. Of 14 responders, 7 (50%) selected “I’ve tested skills for real, and found it helpful”; 4 (29%) chose “I’ve practiced skills but struggled with it”; 2 (14%) selected “I’ve practiced skills, but not used them for real”; and 1 (7%) chose “I’ve not practiced or used skills for real.”

**Table 2:** Engagement rates with value-based goal-setting activities set in sessions 3, 4, and 6.

Goals	Participants who set a goal^a^		Participants who set a behavioral goal^b^			Example participant goal (value)		Participants who met the goal, n (%)^b^			
	N	n (%)	N	n (%)			*Yes*		*No*	*Partly*	Unknown
Goal for day (session 3)	22	20 (91)	20	18 (90)	“Allow my partner to touch my scar.” (intimacy)		10 (50)		3 (15)	5 (25)	2 (10)
Goal for week (session 4)	19	18 (95)	18	16 (89)	“Exercise at the front of gym class.” (courage)		10 (58)		2 (10)	2 (10)	4 (22)
Long-term goal (session 6)	16	5 (33)	5	5 (100)	“Eat out with friends again.” (self-care)		N/A^c^		N/A	N/A	5 (100)^d^

^a^Percentage of participants who set a goal is taken from participants who started the session only.

^b^Percentage of participants who (1) set a behavioral (rather than affective) goal and (2) rated their goal as met are taken from participants who set a goal only.

^c^N/A: not applicable.

^d^Participants were not followed up regarding the outcome of their long-term goal.

### Acceptability

#### Overview

As presented in Table 3, most participants reported satisfactions with the program in terms of comprehensibility, interest, and helpfulness (although a smaller majority reported finding the sessions helpful). Independent samples *t* tests revealed no significant differences in any of the 3 satisfaction ratings between male and female participants or in those with university level of education and high school level education. Pearson correlation coefficients showed no relationship between age and scores on any of the 3 satisfaction ratings.

**Table 3:** Average satisfaction rating scores over all completed sessions (n=30).

Satisfaction ratings	Values, mean (SD)	Scores ≥4 (*somewhat agree*), n (%)
Sessions were interesting	4.28 (0.59)	30 (85.7)
Sessions were easy to understand	4.45 (0.64)	31 (88.6)
Sessions helped me	3.86 (0.76)	21 (60)

Of the 36 participants, 12 (33%) participants took part in exit interviews. The mean age of the interview participants was 39.9 (SD 15.49) years. Of the 12 participants, 10 (83%) were female, 2 (17%) were male, and 11 (92%) were White. Of the 12 participants, 6 (50%) had completed all 6 sessions of the program, and 6 (50%) had not. Thematic analysis of the interviews generated 4 overall themes, with 3 relevant to program acceptability. These are presented in the following sections with illustrative quotes.

#### Mobile Features Facilitated Engagement

Most participants described the program’s mobile features favorably, especially in terms of giving flexible and immediate access:

...it felt like a little pocketbook. And just to sort of pull it out if I was waiting for my daughter to come out of work, I could just read something and it focused my mind a bit.

female, 44 years, acquired craniofacial condition

Participants particularly highlighted the tailored text reminders (simulating notifications) as a crucial feature in prompting the use of the program:

I liked that this alerted you as well, you could set it to send you text messages and it reminded you to be mindful...even though I was busy with work, some of those I set them and they came through during the day and I just did them. Which I thought was super helpful.

female, 26 years, congenital difference

#### Engaging Content (With Room for Improvement)

All participants felt that the multimedia elements of *ACT It Out* aided their understanding of the ACT model, although some felt these elements could be presented in a more dynamic, interactive, and clear way or more material presented visually rather than via text. Some referred to the *toward* and *away* arrows and the *pillars*, and many highlighted the *passengers on the bus* animation:

There’s like a video of the people on the bus...even though I now think about it as maybe too simple, it actually is probably quite effective because I remember it very vividly.

female, 26 years, congenital difference

Some participants described the content as *easy to follow* (male, 21 years, skin condition):

Well I sound strange given I didn’t complete it, but it was still very positive, the bits that I did actually do. It was very clear...the information was very sort of concise and clear.

male, 22 years, skin condition

Others felt improvements could be made to improve the clarity and navigation of the program:

I wasn’t sure to do these [exercises], do they get recorded [in the program] or are you just doing it for yourself? That wasn’t clear enough to me I don’t think.

female, 56 years, acquired craniofacial condition

#### Challenges to Sustained Use

Some participants, including noncompleters, discussed the effort required to fully engage, such as needing strong motivation, self-discipline, and sufficient energy to work through the program in the face of competing demands and stressors:

...with all the apps you’ve got on your phone...it’s massive competition for attention and those other things like Tik Tok and Instagram, they’re obviously geared towards trying to reward. And this [program] requires a lot more self-discipline and it’s something that’s not necessarily going to make you feel good or anything like that.

male, 22 years, skin condition

To place participants’ views in context, it is noteworthy that they highlighted challenging personal circumstances and stressors as a barrier to engaging fully in the course, especially given that the study spanned the COVID-19 pandemic: “...when obviously the pandemic started to happen with the Coronavirus and the lockdown and then things changed at home, I just found it kind of impossible to remember to actually go on it” [female, 24 years, congenital condition].

### Clinical Safety and Preliminary Effectiveness

#### Overview

Table 4 shows the results of the MMRM analyses for all outcome measures. Regarding the primary outcomes, appearance fixing significantly decreased at 8 weeks; however, improvements were lost at 12 weeks, and the opposite was found for life disengagement. Of the 2, only appearance fixing showed significant improvements over the combined time points. Hedge *g* corrected effect sizes for the primary measures ranged between 0.34 and 0.62 at premeasurement to week 8 measurement and premeasurement to week 12 measurement, suggesting small to medium effects. Changes in ACT measures were significantly favorable in all analyses, other than the valued action from premeasurement to week 12 measurement. Of the 36 participants, at baseline, 5 (14%) participants scored in the mild range for depression caseness and 5 (14%) scored in the moderate range; 3 (8%) scored in the severe range for anxiety, 11 (31%) scored in the mild range, and 7 (19%) scored in the moderate range. Overall anxiety and depression scores were significantly reduced, and although anxiety scores did not significantly reduce at week 8, the trend was toward a decrease, suggesting no iatrogenic effects from the program. Floor and ceiling effects were not detected other than for fear of negative appearance evaluation at baseline measurement, in which 17% (6/36) of participants reported the highest possible score, suggesting a higher level of concern and a possible ceiling effect.

Of the 4 themes from the interview data, 1 (25%) related to clinical safety and preliminary effectiveness.

**Table 4:** Descriptive statistics and mixed model repeated measure results for baseline to week 8, baseline to week 12, and combined time effects.

Measures			Baseline, mean (SD)		Week 8, mean (SD)		Week 12, mean (SD)		Baseline-week 8							Baseline-week 12							Combined time effect		
									*F* test (*df*)		*P* value		Hedge *g* (95% CI)^a^		*F* test (*df*)			*P* value		Hedge *g* (95% CI)^a^		*F* test (*df*)			*P* value
**Clinical well-being**																									
	HADS^b^-Anxiety	9.78 (3.67)		8.21 (3.79)		7.59 (3.05)		2.76 (35)		—^c^		0.41 (–0.91 to 0.08)		7.15 (35)			—		0.64 (0.15 to 1.13)		3.66 (35)			.03	
	HADS-Depression	6.33 (3.46)		4.39 (3.43)		4.39 (3.16)		5.02 (35)		.03		0.56 (0.05 to 1.06)		5.80 (35)			.02		0.58 (0.09 to 1.07)		3.67 (35)			.03	
**ACT^d^ measures**																									
	BEAQ^e^	56.83 (10.92)		50.46 (11.90)		49.26 (12.55)		4.85 (35)		.03		0.55 (0.05 to 1.06)		6.84 (35)			.01		0.64 (0.15 to 1.13)		4.23 (35)			.02	
	CFQ^f^	33.03 (6.00)		29.00 (6.10)		28.48 (6.88)		6.96 (35)		.01		0.66 (0.15 to 1.17)		8.17 (35)			.006		0.70 (0.20 to 1.19)		5.37 (35)			.007	
	CompACT-VA^g^	32.31 (7.16)		36.32 (5.98)		32.94 (5.99)		5.97 (35)		.02		0.59 (0.09 to 1.10)		0.15 (35)			—		0.09 (–0.39 to 0.57)		3.59 (35)			.03	
**Appearance**																									
	BILD-Q^h,i^	2.10 (0.58)		1.77 (0.56)		1.87 (0.71)		5.27 (35)		.03		0.57 (0.06 to 1.07)		1.95 (35)			—		0.34 (0.14 to 0.83)		2.76 (35)			—	
	BICSI-AF^i,j^	1.98 (0.59)		1.68 (0.62)		1.60 (0.61)		3.91 (35)		—		0.49 (–0.01 to 1.00)		6.55 (35)			.01		0.62 (0.13 to 1.11)		3.74 (35)			.03	
	FNAES^k^	24.61 (5.49)		19.28 (7.38)		20.00 (6.63)		10.20 (35)		.002		0.82 (0.31 to 1.34)		9.4 (35)			.003		0.75 (0.26 to 1.25)		7.36 (35)			.001	
	BESAA-A^l^	1.12 (0.70)		1.68 (0.91)		1.63 (0.92)		7.23 (35)		.01		0.69 (0.18 to 1.20)		6.43 (35)			.01		0.63 (0.14 to 1.12)		5.18 (35)			.008	

^a^Hedge *g* corrected effect size follows Cohen *d* thresholds of 0.2 to 0.49 for small effects, 0.5 to 0.79 for medium effects, and ≥0.8 and above for large effects [60].

^b^HADS: Hospital Anxiety and Depression Scale.

^c^Not available.

^d^ACT: acceptance and commitment therapy.

^e^BEAQ: Brief Experiential Avoidance Questionnaire.

^f^CFQ: Cognitive Fusion Questionnaire.

^g^CompACT-VA: Comprehensive Assessment of Acceptance and Commitment Therapy–Valued Action subscale.

^h^BILD-Q: Body Image Life Disengagement Questionnaire.

^i^Primary outcome measures.

^j^BICSI-AF: Body Image Coping Strategies Inventory–Appearance-fixing.

^k^FNAES: Fear of Negative Appearance Evaluation Scale.

^l^BESAA-A: Body Esteem for Adolescents and Adults–Appearance subscale.

#### How the Program Helped

Relevant to clinical safety, some participants described the content as supportive and encouraging, especially the interaction from the app guide: “I came out of [a mindfulness exercise], before I read [the app guide’s] comment, thinking ‘Well that was pointless’, but then reading what she said, it was like well actually no, it’s quite difficult to do and I don’t feel so bad about it now” [female, 44 years, acquired craniofacial condition].

Most participants talked about specific ways in which using *ACT It Out* had benefited their behavior and/or self-awareness regarding their appearance. Some referred to ACT-specific content such as value-based goal setting as facilitative of behavior change, whereas others highlighted appearance-specific content such as social skills training or a combination of the 2, such as *safety mode* behaviors like appearance comparisons:

[ACT It Out] actually inspired me to delete Facebook. I did find that it was a massive trigger to me...I think it was the question prompts, when it said about comparing yourself to others, and it really made me think actually I do that a lot of the time. I just thought “I don’t need that in my life. Why am I spending 2 hours scrolling through Facebook, to look at other people that look perfect?”

female, 24 years, congenital condition

Many participants described the course content as bringing about greater self-awareness of psychological processes and their link to overt behavior:

So I have some old habits as a result of my scarring which I didn’t know were there...For example when I go for a run I’ll wear a sports top, and while I’m running I touch my chest. I didn’t know I did that, because I was thinking people are looking at me, so I try and move my top around and things like that, which are a bit odd. And I hadn’t noticed that before until I did the app. I suppose [it was because of] those people when they gave their experiences [in ACT It Out], the little bubbles with personal experiences. That resonated with me.

female, 23 years, skin condition

## Discussion

### Principal Findings

The overall finding of this feasibility study was that a novel prototype mobile ACT program, *ACT It Out*, showed proof of concept for adults with visible differences who experienced appearance concerns. The results pertaining to the primary feasibility objectives of determining user engagement and acceptability support this overall finding.

In terms of user engagement, over half of the participants completed the minimum cutoff of at least 4 of 6 sessions, and full completion rates were at the upper end of the 34% to 41% range reported in a recent meta-analysis on apps for anxiety, depression, and stress, whereas the observed decline in participant adherence over time similarly follows the meta-analytical findings [61]. Combined, these findings suggest acceptable levels of adherence. The finding that higher age significantly predicted a greater likelihood of completion is of potential noteworthiness. Research findings on the influence of age on mental health app adherence are equivocal, with 3 studies included in a meta-analysis finding no relation and 1 finding older age to be associated with greater adherence [61]. Given that in this study, young interview participants made more mention of the look and feel of the prototype than older participants, it may be that the younger participants held higher expectations of the prototype’s interface and were more deterred by the somewhat basic Qualtrics web app interface. This finding could also reflect the overall higher use of smartphone apps in younger people, especially in the form of social, gaming, and video apps [62], which may offer greater competition for smartphone use, as noted by a young noncompleter participant in the interviews. The apparently lower completion rates of male participants, who were also younger on average than female participants, introduce a potential alternative or additive explanation. Either way, this finding points to the importance of us gaining feedback from young and male user representatives when developing a final native app. It also poses the question of whether participants’ use rates of other apps would moderate their level of engagement with the final *ACT It Out*. This could be answered in a larger trial by asking participants to self-report their broader app use. The finding that individuals with a lower level of education were more likely to complete all sessions is also of interest. As part of the stakeholder-informed design phase of *ACT It Out*, the authors made a concerted effort to create an accessible and comprehensible intervention. As reflected by a minority of interview participants, some of the program content may potentially have been considered overly simplistic, and hence less stimulating for some. That being said, there was no difference in participants’ satisfaction ratings of interest between those with university education and those with high school education. Nevertheless, offering optional *advanced* informational resources in a final app would help enrich the depth of material while retaining the intervention’s comprehensibility.

Encouragingly, participants’ average time spent completing sessions was consistent with the design intentions of 30 minutes per session over 6 to 8 weeks, with individual sessions completed in a mixture of single and multiple sittings, according to supplementary interview data. This tallies with the interview findings that participants valued being able to flexibly work through the program according to their schedule. The finding that most participants reported completing mindfulness practices only semiregularly aligns with the literature on mindfulness app use, for example, the study by Mikolasek et al [63]. Mindfulness practice rates may also have been hindered by the web app interface, with some interview participants discussing the challenge of navigating into target content in Qualtrics after receiving text reminders. The same issue may have contributed to the low uptake of long-term value-based goal setting, a feature accessed via *Skill builders* in the program menu rather than in session. Given apparently higher engagement with social skills training and its tangible relevance to appearance concerns, its earlier introduction in the program could potentially improve adherence.

Overall, the program was rated as acceptable, with the percentage of affirmative satisfaction ratings pertaining to comprehensibility and interest well above acceptability thresholds of 70% used in comparable app feasibility studies, for example, the study by Huberty et al [38]. The lower majority of participants rating the sessions as helpful may partly reflect the questions being delivered immediately at the end of each session, thereby giving no practice time for skills introduced in the session.

The interview data offered vital insight into participants’ experience of using the program in terms of its suitability and appeal, highlighting both strengths and areas for refinement. Participants’ accounts endorsed the added value of mobile-specific benefits such as tailored, immediately actionable reminders and remote, location-flexible accessibility. Most interview participants also described the content as appropriately clear, concise, and engaging for the mobile platform, a common challenge in adapting material from other self-help formats [32] (as was involved in the design process of this program). The supportive tone described by participants may reflect the importance placed on this aspect in the program’s design, based on preceding stakeholder-informed design work. The need for personal effort and self-discipline highlighted by some participants may suggest that a greater degree of extrinsic reward be built into future versions of the program. This could include elements successfully used in ACT-based behavior change apps, for example, in the study by Bricker et al [64], such as badges awarded for completed goals and sequential unlocking of features such as mindfulness exercises as users progress.

Results pertaining to the secondary objective of assessing the program’s clinical safety and preliminary effectiveness are only indicative, given the possibility of artifact findings arising from demand characteristics in single-group designs [65], the small sample size, and the short-term measurement. Nevertheless, the finding that all outcomes, including primary appearance-related behaviors and ACT process measures, showed significantly favorable changes from baseline to at least one of the week 8 and week 12 time points offers encouragement for the program’s potential effectiveness under more rigorous evaluation.

Importantly, for the purpose of checking for iatrogenic effects from the program, there was no increase in depression or anxiety over time. Similarly, both secondary appearance-related measures of appearance self-evaluation and fear of negative appearance evaluation, which were not directly targeted through the program’s focus on valued action, improved over the 3 time points. However, the FNAES [47] used did show a signal of a ceiling effect at baseline measurement, with >15% of participants scoring the maximum fear of negative evaluation score. This indicates potential concern with its content validity and responsiveness in the target population [55] and hence requires careful consideration for use in future RCTs. The finding that improvements in valued action were lost at week 12 may be partly explained by the low uptake in long-term value-based goal setting in the final session, suggesting that the design of this feature requires close attention in the future version. Interviews also offered illustrative accounts of specific ways in which participants benefited from the program, most prominently in terms of reducing appearance-fixing behaviors (eg, engaging in hours of appearance comparisons on Facebook and habitually adjusting sportswear).

### Strengths and Limitations

A strength of the study is the comprehensive and stakeholder-focused process through which the program, *ACT It Out*, had been designed with user representatives and specialist clinicians. It is also both the first published ACT-based self-help intervention and the first mobile intervention to have been tested for adults with visible differences. The mixed methods methodology and addition of the semistructured qualitative interviews, in particular, provided useful insight into participants’ experiences to inform further development not otherwise captured through data use and self-report ratings.

The limitations of this study include its co-occurrence with the COVID-19 pandemic and the potential for confounding effects on at least some data. Although data were collected during a period of lesser restrictions, the context of participants’ lives was nevertheless altered in ways relevant to common appearance concerns in individuals with visible differences. For example, the implementation of mandated mask-wearing may have offered individuals with facial differences such as cleft lip and/or palate a socially sanctioned means of concealment, and social restrictions may have similarly reduced some participants’ appearance concerns because of enforced minimization of social contact. Conversely, the widespread use of video calling and conferencing platforms during the pandemic has been indicated as a source of heightened appearance anxiety for individuals with visible differences (personal communication by Professor Diana Harcourt, October 14, 2021). Therefore, the exact impact of the pandemic context on participants’ data is difficult to determine. Nonetheless, the validity and reliability of certain outcome measures such as disengagement with appearance-salient activities may have been adversely affected.

The widespread disruption caused by the pandemic may also at least partly account for participants’ higher-than-expected anxiety scores at baseline, as suggested by an increase in anxiety scores since the pandemic in the general UK population [66]. Some noncompleter participants also cited COVID-19–related disruption as a cause of program nonadherence in interviews, suggesting that adherence rates may have been higher under typical circumstances. Other data more centered on the content of *ACT It Out* and the experience of using it, such as satisfaction ratings and the detailed accounts collected via interviews, should be less dependent on societal conditions.

Although the sample covered a wide range of appearance-affecting conditions and ages, participants were predominantly White females, and scarring was underrepresented in the sample, limiting the study’s generalizability to the visibly different population. The self-selecting nature of the interview subsample may also limit the validity of the interview findings, although half of the subsample were noncompleters, mitigating the potential for positivity bias.

### Conclusions

Despite these limitations, overall findings suggest promising feasibility of the *ACT It Out* program via adequate levels of engagement, acceptability, and indication of clinical safety and positive changes in outcomes. The study also yielded valuable direction for refinements to further enhance its potential utility. When developed further and shown to be effective under more rigorous evaluation, the program could offer a valuable standalone resource for individuals with visible differences who have mild to moderate appearance concerns, as well as a tool for specialist clinicians to use alongside psychological therapy.

## Abbreviations

ACT: acceptance and commitment therapy
BICSI-AF: Body Image Coping Strategies Inventory–Appearance-fixing
CFQ: Cognitive Fusion Questionnaire
FNAES: Fear of Negative Appearance Evaluation Scale
HADS: Hospital Anxiety and Depression Scale
MMRM: mixed model repeated measure
RCT: randomized controlled trial
